# Opposite effect of Valproate on Tax and HBZ expression in T-lymphocytes from HTLV-1 asymptomatic carriers and HAM/TSP patients

**DOI:** 10.1186/1742-4690-8-S1-A198

**Published:** 2011-06-06

**Authors:** Gildas Belrose, Antoine Gross, Stéphane Olindo, Agnès Lézin, Maryvonne Dueymes, Didier Smadja, Yuetsu Tanaka, Luc Willems, Jean-Michel Mesnard, Jean-Marie Peloponese, Raymond Césaire

**Affiliations:** 1 Laboratoire de Virologie–Immunologie, EA 4537, Centre Hospitalier Universitaire de Fort-de-France, Fort-de-France, Martinique; 2 CEAPB, CNRS UMR 5236, Université de Montpellier, Montpellier, France; 3 Service de Neurologie, EA 4537, Centre Hospitalier Universitaire de Fort-de-France, Fort-de-France, Martinique; 4 Department of Immunology, Graduate School and Faculty of Medicine, University of the Ryukyus, Okinawa, Japan; 5 Cellular and Molecular Biology, Agro-Bio Tech, Gembloux, Belgium

## 

A determinant of HTLV-1 associated myelopathy/tropical spastic paraparesis (HAM/TSP) development is HTLV-1-infected cell burden. Viral proteins Tax and HBZ, encoded by the positive and negative strands of the pX region respectively, play a key role in HTLV-1 persistence. Tax drives CD4+ T-cell clonal expansion but is the immunodominant antigen. Valproate (VPA), an histone deacetylase inhibitor, has been proposed to trigger Tax expression and expose latent HTLV-1 reservoir to immune destruction. We evaluated the impact of VPA treatment on Tax, Gag and HBZ expression in cultured lymphocytes from asymptomatic HTLV-1 carriers and HAM/TSP patients. Around one-fifth of provirus-positive CD4+ T cells spontaneously became Tax-positive. The estimation rose up to two-thirds of Tax-positive infected cells when VPA was added. VPA enhanced Gag p19 protein release. Tax and Gag mRNA levels spontaneously peaked, before a decline concomitant to HBZ mRNA increase. VPA treatment enhanced and prolonged Tax mRNA expression, while it blocked HBZ expression. This is the first ex vivo study on the balance between Tax and HBZ expression (i.e. sense and antisense transcription). Our data suggest that besides modulation of the expression of Tax, another mechanism involving repression of HBZ may determine the outcome of VPA treatment on HTLV-1-infected cell proliferation and survival.

